# Prevalence and Pattern of Eye Disorders Among Primary Schoolchildren in Abakaliki, Nigeria: A Cross-Sectional Study

**DOI:** 10.7759/cureus.53385

**Published:** 2024-02-01

**Authors:** Dilichukwu I Aniemeka, Edak Ezeanosike, Chinenyenwa Okanya, Onyekachi J Ireka, Chimdia E Ogbonnaya, Azuka S Adeke, Amarachi N Onyebuchi

**Affiliations:** 1 Department of Ophthalmology, Alex Ekwueme Federal University Teaching Hospital, Abakaliki, NGA; 2 Department of Community Medicine, Alex Ekwueme Federal University Teaching Hospital, Abakaliki, NGA; 3 Department of Radiology, Alex Ekwueme Federal University Teaching Hospital, Abakaliki, NGA

**Keywords:** schoolchildren, vision screening for schoolchildren, pattern of ocular morbidity, prevalence of ocular morbidity, school eye health, eye disorders

## Abstract

Background

A child’s learning ability depends on vision, and visual impairment negatively affects neurological, intellectual, and emotional development by limiting children’s exposure to a range of experiences and information. This study aims to determine the prevalence and pattern of ocular morbidity among primary schoolchildren in Abakaliki and provide evidence that can be used in planning a school eye health program for the state.

Methodology

A school-based, cross-sectional study was conducted within the Abakaliki metropolis between January and April 2018 to determine the ocular health status of schoolchildren recruited using stratified random sampling. Data were analyzed using SPSS version 22 (IBM Corp., Armonk, NY, USA) and presented using descriptive statistics. Univariate analysis was performed to determine the association between dependent and independent variables, with the level of significance determined by a p-value <0.05 (95% confidence interval (CI)).

Results

A total of 553 schoolchildren aged 6-16 years were examined. The prevalence of eye disorders was 23.5%. Common disorders included refractive error (12.7%) and allergic eye disease (7.1%). Other findings included glaucoma suspect (15, 2.7%), infective conjunctivitis (1, 0.2%), amblyopia (3, 0.5%), cataract (1, 0.2%), and squint (1, 0.2%). The prevalence of visual impairment was 3.1%. Univariate analysis showed a significant association between ocular morbidity and attending private schools (95% CI = 6.5-11.1, p = 0.003).

Conclusions

Eye disorders such as uncorrected refractive error and allergic conjunctivitis were common among schoolchildren. School eye health programs can ensure that eye screening is done periodically, allowing for early detection, referral, and prompt treatment of eye diseases that can potentially cause visual impairment.

## Introduction

A child’s learning ability depends on vision. Vision development is critical between the ages of 0 and 12 years, with visual impairment negatively affecting neurological, cognitive, and emotional development by limiting children’s exposure to a range of experiences and information [1]. According to the World Health Organization (WHO), up to three-quarters of all blindness worldwide is avoidable, and about one-half of the causes can be prevented or treated [2].

In Nigeria and elsewhere, primary schoolchildren form an important, large target group that should be screened adequately to detect eye diseases early and prevent blindness. This is because children who become blind can potentially live with this disability for many years, which may significantly impact their educational outcomes and socioeconomic development [3,4]. In low-resource settings such as ours, school-aged children may be affected by several eye diseases, including refractive error, trachoma, conjunctivitis, allergic eye diseases, injuries, amblyopia, lid disorders, squint, vitamin A deficiency, and congenital cataracts [5,6]. Some studies conducted in Nigeria have recorded the prevalence of visual impairment among schoolchildren ranging from as low as 1% to as high as 26.1% [7-9]. Due to a lack of appropriate eye healthcare, many children may develop permanent vision loss. However, there is little evidence in Abakaliki to demonstrate the significance of school eye health programs.

Despite the recommendation by the Federal Ministry of Education, Nigeria, to conduct periodic eye screening of schoolchildren, as detailed in the school health policy, implementation is lacking. Therefore, determining the ocular health status of these children will not only provide data for effective planning of school eye health programs but will also reiterate the need to implement the policy to prevent blindness, visual impairment, poor school performance, and drop-outs through early detection and treatment [10].

This study aims to determine the prevalence and pattern of ocular morbidity among primary schoolchildren in the Abakaliki metropolis and provide data for effective planning of school eye health programs in the state.

## Materials and methods

Pilot study

A pilot study was conducted in two schools selected randomly from Ohaukwu L.G.A. outside the Abakaliki metropolis to test the adequacy of the research instrument and the feasibility of the study, standardize the study definitions, and modify the data collection tools. Research assistants were observed during the study procedure to ensure the uniformity of the results.

Study design

This cross-sectional study was conducted to determine the prevalence and pattern of eye disorders among primary schoolchildren in Abakaliki.

Study setting

The study was conducted in selected schools located in the Abakaliki metropolis of Ebonyi State, in the southeast geopolitical zone of Nigeria, between January and April 2018. Abakaliki metropolis has a population of 915,438 and comprises the Abakaliki and Ebonyi Local Government Areas [11].

According to the data obtained from the Ebonyi State Ministry of Education and Universal Basic Education Board [12], 21,090 schoolchildren attended 56 registered primary schools located in the Abakaliki metropolis.

A total of 553 participants included in the study were randomly selected primary schoolchildren aged 6-16 years who attended primary schools within the Abakaliki metropolis of Ebonyi State and were cooperative during the examination.

The independent variables studied included sociodemographic factors such as age, gender, and type of school attended, while the dependent variable was the prevalence and pattern of eye disorders such as allergic conjunctivitis, refractive error, glaucoma suspect, cataract, and amblyopia, as determined by the study definitions.

Diagnostic criteria

A glaucoma suspect was defined as one of the following findings in at least one eye [13]: (a) an optic nerve or nerve fiber layer defect suggestive of glaucoma (≥0.5 cup-disc ratio, asymmetric cup-disc ratio of ≥0.2, notching or narrowing of the neuroretinal rim, a disc hemorrhage, or suspicious alteration in the nerve fiber layer); (b) a visual field abnormality consistent with glaucoma; and (c) an elevated intraocular pressure greater than 21 mmHg.

Amblyopia was defined as the unilateral or, rarely, bilateral, decrease in best-corrected visual acuity (VA) or a difference in best-corrected VA of two Snellen lines or more (or >1 log unit) caused by form vision deprivation and/or abnormal binocular interaction, for which there is no identifiable pathology of the eye or visual pathway [14].

Myopia was defined as ≥-0.50 DS, hypermetropia as ≥+0.50 DS, and astigmatism as ≥+0.50 Dcyl or −0.50 Dcyl.

Allergic conjunctivitis was defined based on any one of the following: a history of itching and burning, redness or brownness, lacrimation, photophobia, signs of a mucinous, ropy discharge, and/or clinical presence of two or more papillae in the lower or upper tarsal conjunctiva [15].

Using Oyedeji’s classification of socioeconomic status, the pupils were classified into upper, middle, and lower socioeconomic status [16]. The International Classification of Diseases 11 Revision (2018) was used to classify vision distance presenting vision impairment [17]: mild - presenting VA worse than 6/12; moderate - presenting VA worse than 6/18; severe - presenting VA worse than 6/60; and blindness - presenting VA worse than 3/60.

The data collection tool was developed by the research team, adapted, and modified to suit the objectives of the research.

Data sources/measurements

Randomly selected students who met the inclusion criteria were given identification numbers. The nurse filled out the biodata of the child and measured his/her unaided/aided distant VA in a well-illuminated room at a distance of 6 m. A pinhole VA was done in children with VA ≤6/9. If VA improved with pinhole, the child was sent to the trainee opthalmologist for refraction and ocular examination. However, if the VA did not improve with the pinhole, the child was sent to the principal investigator (ophthalmologist) for further ocular examination.

Objective and subjective refraction was performed for children with VA <6/6 that improved with pinhole after ocular examination by the principal investigator to rule out other ocular pathology.

Ocular examination comprised anterior-segment examination using a pen torch and head loupe and posterior-segment examination with a direct ophthalmoscope. Cover test and Hirschberg test were done to assess phorias and tropias.

Children with a vertical cup-to-disc ratio of ≥0.5 and/or disc asymmetry of ≥0.2 in the measurement of the vertical cup-to-disc ratio between the two eyes, or other disc abnormality suggestive of glaucoma, had their intraocular pressure measured using a Perkins tonometer. They were also subsequently referred to the Federal Teaching Hospital Abakaliki for a more detailed examination. All findings were noted on the Refractive Error Study in Children eye examination form.

Eye drops (guttae Occullerg) were given to pupils who had allergic conjunctivitis. Other cases of ocular morbidity were referred for further management at the Federal Teaching Hospital.

Parents and guardians of all referred cases were contacted, and reminders were sent to them for further examinations.

Study size/Sampling method

The sample size was determined using the following formula: N = Z2PQ/d2, with a 95% confidence interval (CI) and a degree of accuracy of 5%. We accounted for design effect due to cluster sampling (1.5) and a 10% attrition rate to arrive at a sample size of 553.

Using the multi-stage random sampling of a computer-generated set of numbers, five public schools and six private schools were included in the study.

Using simple proportion, 553 children to be examined were allocated to the 11 schools selected. At the school level, each class grade was taken as a cluster, with every school having six clusters (classes 1-6). Using a simple random sampling method, the children to be examined were selected by generating a set of numbers using Microsoft Excel (Microsoft Corp., Redmond, WA, USA). The children who were randomly selected from the register were identified and examined after ensuring they had a consent form duly signed by their parents or guardians. The examination was conducted by two teams, with each team consisting of one ophthalmologist, a trainee ophthalmologist, one optometrist, and one ophthalmic nurse.

Statistical analysis

Data cleaning was done by the principal researcher daily and entered in an Excel sheet while coding and analyses were performed using SPSS for Windows version 22 (IBM Corp., Armonk, NY, USA). The sociodemographic characteristics of the study participants were analyzed using descriptive statistics. Continuous variables were summarized as mean and standard deviation, while categorical variables were described as percentages and proportions. The chi-square test of statistical significance was used in the analysis, and the level of statistical significance was determined by a p-value of <0.05 at a 95% CI.

Ethical considerations

Ethical approval was obtained from the Health Research and Ethics Committee of Federal Teaching Hospital Abakaliki (approval number: FETHA/REC/VOL1/2017/615). The study conformed to the Declaration of Helsinki.

Written informed consent was obtained from the pupils, duly signed by their parents/guardians. It included the purpose of the study and further explained that the data collected was confidential. The collected data was stored in a password-protected computer.

## Results

Sociodemographic characteristics of participants

A total of 553 primary school pupils (315 females and 238 males) were examined. The mean age of the children was 9.8 ± 2.1 (age range = 6- 16 years), with a modal age of nine years. There were more females than males with a male-to-female ratio of 1:1.3. The majority (67.8%) of the children were from the middle socioeconomic class. Moreover, 16.5% of the children had symptoms of eye disorder (Table 1).

**Table 1:** Sociodemographic characteristics of the pupils studied.

Variables	Private school (274)	Public school (279)	Total pupils (553)
	n (%)	n (%)	n (%)
Age (year)			
6–9	137 (50.0)	138 (49.5)	275 (49.7)
10–13	134 (48.9)	132 (47.3)	266 (48.1)
14–16	3 (1.1)	9 (3.2)	12 (2.2)
Sex			
Male	103 (37.6)	135 (48.4)	238 (43.0)
Female	171 (62.4)	144 (51.6)	315 (57.0)
Socioeconomic distribution			
High	16 (5.8)	4 (1.4)	20 (3.6)
Middle	216 (78.8)	159 (57.0)	375 (67.8)
Low	42 (15.4)	116 (41.6)	158 (28.6)
Pupils with ocular complaints	62 (22.6)	29 (10.4)	91 (16.5)

Descriptive analysis

Using presenting VA, the majority of the pupils examined had normal vision in both the right and left eye (96.9% and 97.1%, respectively). According to the WHO ICD-11 classification of visual impairment, mild visual impairment was the most common form of visual impairment noted in the study population. Moreover, 0.2% had unilateral blindness as a result of congenital cataracts (Table 2).

**Table 2:** Prevalence of visual impairment among the primary schoolchildren in the Abakaliki metropolis.

Visual acuity		Right eye (n = 553)		Left eye (n = 553)	
		Frequency	%	Frequency	%
6/5–6/6		480	86.8	487	88.1
<6/6–6/9		34	6.1	31	5.6
<6/9–6/12		22	4.0	19	3.4
<6/12–6/18	Mild visual impairment	8	1.4	8	1.4
<6/18–6/60	Moderate visual impairment	7	1.3	4	0.7
<6/60–3/60	Severe visual impairment	2	0.4	3	0.5
<3/60	Blindness	0	0	1	0.2

Symptoms of eye disorders among schoolchildren

**Figure 1:**
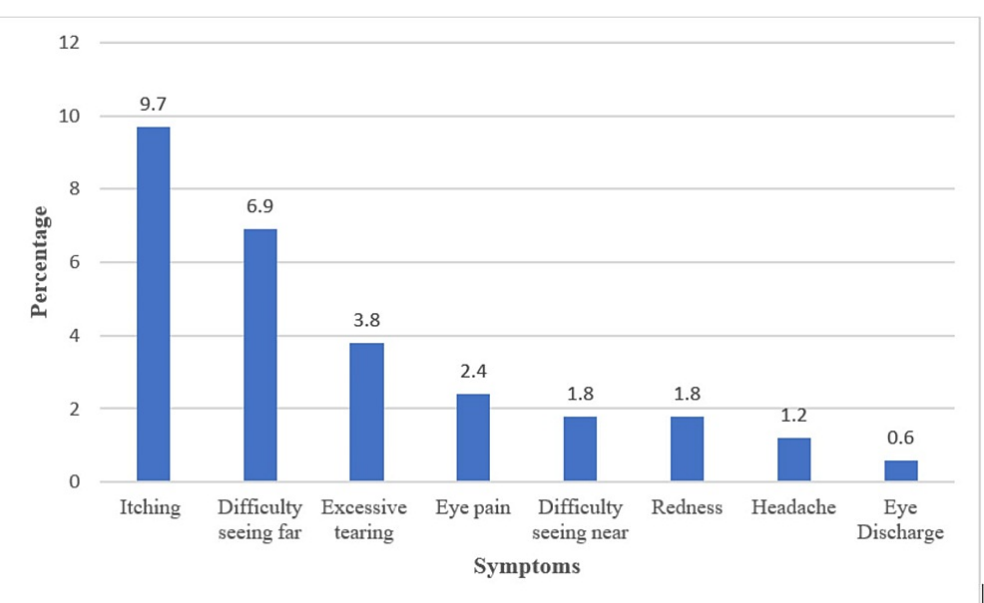
Symptoms of eye disorder among the schoolchildren (%).

The most common complaints among the pupils in this study were itching (9.7%) and difficulty seeing far (6.9%); however, few pupils complained of excessive tearing (3.8%), eye pain (2.4%), and headache (1.2%) (Figure 1).

Pattern and prevalence of eye disorders among schoolchildren

Table 3 shows the pattern of ocular morbidity among primary schoolchildren in private and public schools. The prevalence of ocular morbidity was 23.5%, and the most common ocular morbidity was refractive error followed by allergic conjunctivitis. There was a higher prevalence of ocular morbidity among children attending private schools compared to children attending public schools within the Abakaliki metropolis. Pearson’s correlation analysis indicated a strong association between children attending private schools and an increased prevalence of ocular morbidity (p ≤ 0.003, 95% CI = 6.5-11.1).

**Table 3:** Pattern of ocular morbidity among the primary schoolchildren in the Abakaliki metropolis.

Ocular morbidity	Frequency (%)	Private	%	Public	%	P-value
Refractive error	70 (53.8)	44	33.8	26	20.0	0.003
Allergic conjunctivitis	39 (30.0)	29	22.3	10	7.7	
Glaucoma suspect	15 (11.5)	8	6.1	7	5.4	
Amblyopia	3 (2.3)	1	0.8	2	1.5	
Bacterial conjunctivitis	1(0.8)	1	0.8	0	0.0	
Developmental cataract	1 (0.8)	0	0.0	1	0.8	
Strabismus	1 (0.8)	1	0.8	0	0.0	
Total	130 (100)	84	64.6	46	35.4	

Univariate analysis (correlation between eye disorders and age/gender among schoolchildren)

The frequency of eye disorders was higher in females compared to males and in children aged 10-13 years old. However, there was no statistically significant relationship between ocular morbidity and gender and age (p = 0.899 and p = 0.791, respectively) (Table 4).

**Table 4:** Correlation between the prevalence of ocular morbidity and age and gender.

	Gender (p = 0.899)		Age (years) (p = 0.791)		
Ocular morbidity	Male, n = 52 (40%)	Female, n = 78 (60%)	6–9, n = 60 (46.2%)	10–13, n = 59 (45.4%)	14–16, n = 11 (8.4%)
Refractive error	28 (21.5)	42 (32.3)	27 (20.8)	35 ( 26.9)	8 (6.1)
Allergic conjunctivitis	10 (7.7)	29 (22.3)	21 (16.2)	17 (13.1)	1 (0.8)
Glaucoma suspect	10 (7.7)	5 (3.8)	8 (6.1)	5 (3.8)	2 (1.5)
Amblyopia	2 (1.5)	1 (0.8)	2 (1.5)	1 (0.8)	0 (0.0)
Bacterial conjunctivitis	0 (0.0)	1 (0.8)	1 (0.8)	0 (0.0)	0 (0.0)
Cataract	1 (0.8)	0 (0.0)	1 (0.8)	0 (0.0)	0 (0.0)
Strabismus	1 (0.8)	0 (0.0)	0 (0.0)	1 (0.8)	0 (0.0)

The frequency of ocular morbidity was higher in children from the middle socioeconomic status, and refractive error was the most common ocular morbidity in this group. However, there was a weak correlation between the prevalence of ocular morbidity and socioeconomic status (p = 0.71).

## Discussion

Sociodemographics

In this study, the age range of the primary schoolchildren was 6-16 years which is wider than the expected age range according to the National Policy on Education in Nigeria [18]. This can be explained by increased enrolment into primary schools because of free education provided for basic education; therefore, children from low-income families attend school, even at an older age. This trend was observed in some other studies among primary schoolchildren in Ghana [19], as well as in studies by Okoye et al. [20], and Ogbonnaya et al. [21].

The majority of children were from the middle socioeconomic status, using occupation as an indicator [16], and more than two-thirds of ocular morbidity was seen among this group. This study did not find a correlation between socioeconomic status and ocular morbidity which differs from observations in studies from Nigeria [22], Saudi Arabia [23], and India [24] where the prevalence of ocular morbidity was found higher in children from low socioeconomic backgrounds. Parents in the middle socioeconomic status were mostly civil servants who work from 8 am to 4 pm daily; therefore, it may be difficult to seek eye care services for their wards during workdays.

Symptoms

Itching and difficulty seeing far were the most common complaints among these children. These findings are contrary to the study from Mangalore [4] conducted among pupils aged 10-13 years, where the majority (24.6%) of pupils complained of headaches. However, these findings are comparable to other studies from Ghana [19] and Nigeria [22]. Most children may have presented with itching because this study was conducted during the dry season.

Noticeably, about one out of ten children who had ocular morbidity did not have visual-related complaints. This agrees with findings from studies in Ghana and Nigeria [19,22], where few participants were not aware that they had any ocular pathology. For this reason, visual disorders that need intervention may go unnoticed and lead to visual impairment or blindness. This makes it essential that eye diseases among this age group should be routinely sought by conducting eye screening in schools.

Prevalence and pattern of ocular morbidity

The overall prevalence of ocular morbidity among the primary schoolchildren in this study was 23.5%, and similar findings were reported in studies conducted in Mangalore [4], Ilorin [25], Zaria [26], and China [27]. On the contrary, the study by Okoye et al. [20] recorded a lower prevalence of ocular morbidity (6.1%), which may be because the study participants were primary schoolchildren in a rural area and the study was conducted at a different time of the year. The frequency of ocular morbidity was also more common among females compared to studies from Mangalore [4] and Zaria [26], where ocular morbidity was more common in males. The female preponderance in this study could be the reason for this finding.

The prevalence of refractive error found in this study was higher when compared to the prevalence recorded in other studies from the rural areas of Ebonyi (0.9%) [21], and Bayelsa (2.2%) [28]. The children living in urban settlements may be exposed to more near work when compared to children in the rural areas contributing to a higher occurrence of refractive error in the study population. This is evident in studies from India [24] and China [27] where a relatively higher prevalence of refractive error was found. The proportion of children who had various forms of refractive error (hypermetropia, myopia, and astigmatism) is similar to findings from studies in Saudi Arabia and western India [23,29], where hypermetropia was the most common form, and contrary to findings from India [30] and Ghana [31], where myopia as more common in the population studied. The observation in the later studies may be due to the difference in ethnicity, environmental factors, and the age of the study population.

Allergic conjunctivitis was the second most common cause of ocular morbidity in this study. This is similar to the findings of other studies from Nigeria [25] and India [4,29]. In Saudi Arabia [23], lower values were recorded, which may be attributed to the study being a retrospective, hospital-based study. A higher prevalence of allergic conjunctivitis was found in studies from Enugu (48,1%) [20] and southwestern Nigeria (17.8%) [22] conducted in rural areas. The discomfort from excessive itching, pain, and tearing may be linked to increased absenteeism from school. Early diagnosis and treatment are necessary to alleviate symptoms and prevent complications following chronicity.

A small proportion of the children studied were glaucoma suspects. This is similar to findings from the Cross River State [3] and Saudi Arabia [23]. Advocacy for early detection by an eye specialist is necessary to prevent irreversible visual loss.

One case of bilateral developmental cataract was found. The prevalence of cataracts in this study was similar to a study conducted in southwestern Nigeria [22] among a larger population of schoolchildren in a rural area. In contrast, a study conducted in southeastern Nigeria revealed a higher prevalence of 21.7% among children attending special schools; however, a smaller sample size was examined [32]. This shows that many children who have treatable eye conditions such as cataracts are sent to special schools without appropriate management.

Prevalence of visual impairment

The prevalence of visual impairment among primary schoolchildren in this study was 3.1%, with almost half (47.1%) having mild visual impairment. This prevalence is higher than reports from some other parts of Nigeria [9,22] but lower than findings in a study from Edo State [8]. However, some variation was noted in the sample size and methodology such as using best-corrected VA in calculating the prevalence of visual impairment in the study [9, 22]. The major cause of visual impairment was refractive error (88.2%) which is similar to the findings from Ilesha [22] and Zaria [26]. Other causes of visual impairment in this study were amblyopia (0.5%), squint (0.2%), and cataracts (0.2%). These findings differ from other studies [22,31] that found other causes of visual impairment such as corneal opacity, retinal diseases, and vitamin A deficiency.

Study limitations

As dilated fundoscopy was not done, some posterior-segment lesions might have been missed. Moreover, because this study was conducted in primary schools in an urban setting, the results may not be generalizable to a rural setting. Furthermore, cycloplegic refraction was not done, which would have underestimated the prevalence of uncorrected refractive error.

## Conclusions

This study shows that the prevalence of ocular morbidity was high among primary schoolchildren in Ebonyi State, with refractive error being the most common and the leading cause of visual impairment. There is a need for routine eye screening of school-age children, which should be performed yearly in schools by eye care professionals for early detection of ocular morbidity. Implementing school eye screening and providing affordable spectacles for pupils with uncorrected refractive errors should also be promoted.
